# Scale-up approach for supercritical fluid extraction with ethanol–water modified carbon dioxide on *Phyllanthus niruri* for safe enriched herbal extracts

**DOI:** 10.1038/s41598-021-95222-0

**Published:** 2021-08-04

**Authors:** Norsyamimi Hassim, Masturah Markom, Masli Irwan Rosli, Shuhaida Harun

**Affiliations:** Department of Chemical and Process Engineering, Faculty of Engineering and Built Environment, Universiti Kebangsaan Malaysia, 43600 UKM Bangi, Selangor Malaysia

**Keywords:** Chemical engineering, Toxicology

## Abstract

Scaling-up supercritical fluid extraction (SFE) for the extraction of bioactive compounds from herbal plants is challenging, especially with the presence of alcohol-water as co-solvent. Hence, the main objective of this study is to validate the scale-up criteria of SFE process for *Phyllanthus niruri* (*P. niruri*), and analyse the extract safety and profitability process at the industrial scale. The study was performed by using supercritical carbon dioxide (SC-CO_2_) with ethanol–water co-solvent at two operating conditions (L1: 200 bar, 60 °C and L2: 262 bar, 80 °C). The solvent-to-feed ratio (S/F) scale-up validation experiments were conducted at both operating conditions with feed mass capacity of 0.5 kg. The extraction yields and overall extraction curves obtained were almost similar to the predicted ones, with error of 5.13% and 14.2%, respectively. The safety of scale-up extract was evaluated by using a toxicity test against zebrafish embryo (FETT). The extract exhibited a low toxic effect with the LD_50_ value of 505.71 µg/mL. The economic evaluation using a detailed profitability analysis showed that the SFE of *P. niruri* was an economically feasible process, as it disclosed the encouraging values of return on investment (ROI) and net present values (NPV) for all scale-up capacities.

## Introduction

*Phyllanthus niruri* (*P. niruri*) is a species from the *Phyllanthus* genus and Euphorbiaceae family that is widely distributed in tropical and subtropical areas. It is well-known as ‘*dukung anak*’ in Malaysia. This plant was selected for this study due to the bioactivity of its extracts, which was used traditionally to treat constipation, bronchitis, jaundice, diabetes, asthma, ulcer and wound healing^1–4^. *P. niruri* had been widely studied for pharmacological and clinical purposes. Some activities were assigned to the extracts and the compounds isolated from this plant, such as antioxidant^5,6^, antiviral^7,8^, anti-inflammatory^9,10^, hepato-protection and hypotensive^7,11^, antidiabetic^3^, antifungal^12^ and anti-parasitic^13^.

Since 1992, tannins were progressively reported from the genus *Phyllanthus*^14^, in which ellagitannins were the largest group of hydrolysable tannins. The main hydrolysable tannins isolated from *P. niruri* were corilagin, geraniin, gallic acid, and ellagic acid, which are reportedly responsible for antioxidant, anti-inflammatory, antiviral, and antidiabetic activities^3,10,15,16^. Given the importance of hydrolysable tannins from *P. niruri* as bioactive compounds, appropriate techniques for their extraction are required. In general, supercritical fluid extraction (SFE) technology had proved its feasibility with multiple advantages in natural product processing. If compared to the conventional extraction, it gave a shorter extraction time, higher selectivity and utilise less toxic solvents. Other advantages were simple separation of solvent from the final extract and the use of moderate temperatures in the extraction process, hence thermal degradation could be avoided.

Supercritical CO_2_ had been applied to extract bioactive compounds from leaves of *P. niruri*, along with the presence of ethanol–water co-solvent in previous studies^17–19^. However, the development of this technology at industrial level was quite complicated due to the interaction of numerous parameters, whereby the scale-up process involved a few restrictions and difficulties^20^. Besides, the design process for a SFE unit at industrial level could not be fully dependent on the laboratory-scale data, whereby it was very important to cautiously consider the mass transfer parameters. Therefore, a few approaches, such as dimension/geomtery analysis, similarity theory or mathematical modelling had been applied to predict the extraction process at a larger scale with the purpose of design process parameter determination^21^.

According to Prado et al.^22^, results for larger scale process mimicked the results of laboratory-scale in any form of scale-up criteria. Hence, the kinetic study for SFE process by using mathematical modelling on overall extraction curve (OEC) of laboratory-scale data is very crucial. However, many cases on increasing the scale from laboratory to the industrial level showed a significant decrease in extraction yield^23,24^. Hence, intermediate-scale experiment (pilot scale) was a better strategy because it considered the restriction that might occur on the industrial-scale. This way, the laboratory-scale data could be safely utilised to develop the economic evaluation for the SFE process, in which the process tendency is to sustain the extraction yield with increasing scale. According to Pereira et al.^25^, the most reported drawback of SFE for the past 20 years, was high initial investment cost on industrial plants. SFE was considered as too expensive by many investors because of the high investment costs compared to conventional low-pressure equipment. Therefore, the use of this technology for high-added-value products was restricted^26^. However, if considering the use and quality of its product, the operating costs of SFE were relatively low.

Although the research on herbal plants by SFE for different scales were actively studied, there were still concerns on the use and benefits of the plant extracts, especially their safety to human consumption. Therefore, to increase the product’s market potential, the prevention of their potential harmful effects by using toxicity testing is very crucial as it can reveal several hazards to human through laboratory animals. Spulber et al.^27^ demonstrated that the utilisation of rats and mice could be replaced by zebrafish as an alternative model organism. They found that zebrafish and mice showed similar core features of behavioural alterations after developmental exposure to a toxicant. Furthermore, the use of zebrafish had appeared to be cost saving as it could reduce the use of mammals^28,29^. It was also an ideal experimental model by using animal for large-scale research on vertebrate neurodevelopment and behaviour^30,31^.

To date, no study has yet to be conducted on the profitability of SFE process with ethanol–water co-solvent for *P. niruri* at different feed mass capacities. It was predicted that industrial-scale SFE could be developed for the separation process of natural products from this plant at a competitive cost. Therefore, the aim of this study was to validate the best scale-up criterion and perform the profitability analysis for the SFE process of *P. niruri* at a large-scale SFE units. The toxicology of the extract is also conducted by using *Danio rerio* (zebrafish) embryos to evaluate the product safety.

## Materials and methods

### Sample preparation

Dried *P. niruri* samples were obtained from a local herb supplier (HERBagus Trading Sdn. Bhd., Malaysia). The samples were ground into size distributions of 0.3–0.5 mm for laboratory-scale experiment and 0.5–3 mm for scale-up verification experiment. Density of the sample (*ρ*_*s*_) was determined by using Ultra Pycnometer 1000 (Quantachrome Instruments, USA). The samples were stored in a dark cold room for further use. Table 1 showed the sample density, bulk density and porosity value for both sample size distributions.

**Table 1:** Sample density, bulk density and porosity for *P. niruri*.

Particle size (mm)	Sample density *ρ*_*s*_ (kg/m^3^)	Bulk density, *ρ*_*b*_ (kg/m^3^)	Porosity, *ε*
0.3–0.5	1435.13 ± 3.88	260.16 ± 3.13	0.82
0.5–3	1275.77 ± 3.88	177.48 ± 2.45	0.86

± Standard deviation for three replicates.

The laboratory-scale extraction was conducted by using samples with particle size of 0.3–0.5 mm*.* Meanwhile, the scale-up validation extraction was conducted by using samples with larger particle sizes (0.5–3 mm) to avoid channelling effect. According to Hassim et al.^32^, the diffusional mechanism (solid phase) was less representative than the convection (fluid phase) on the extraction process of *P. niruri*. Hence, the size distribution or particle size were assumed to not much affecting the scale-up process. Furthermore, the porosity value for both sample size distributions were not much different as shown in Table 1.

### Supercritical fluid extraction (SFE)

#### Laboratory-scale experiment

For laboratory-scale experiment, an in house-built SFE system equipped with a 25 mL extraction vessel was employed to determine the OEC of 5 g dried and ground *P. niruri* samples. The solvent used in the extraction was carbon dioxide (99.7%), from Alpha Gas Malaysia Sdn. Bhd., along with ethanol–water as co-solvent. Two operating conditions were used, which were centre point (L1) and optimum point (L2) from a previous study on the optimisation of SFE from *P. niruri* by using the Response Surface Methodology (RSM)^33^. In the previous study, pressure, temperature, ethanol–water ratio and co-solvent concentration were the optimised parameters. It was reported that maximum extraction yields and bioactive compounds were obtained from both operating conditions. The process parameter for L1 and L2 were presented in Table 2. The total flow rate was kept constant as it was the basis parameter in determining the CO_2_ and co-solvent concentrations in terms of flow rate.

**Table 2:** Parameters used for L1 and L2 operating conditions.

Parameter	Operating condition	
	L1	L2
Pressure, *P*	200 bar	262 bar
Temperature, *T*	60 °C	80 °C
Co-solvent type	50% ethanol in water	30% ethanol in water
Co-solvent concentration	10% (v/v)	13% (v/v)
Total flow rate, *f*	1.5 mL/min	1.5 mL/min

Static extraction was conducted for one hour, followed by four hours of dynamic extraction, whereby the extracts were collected every 30 min. The collected extracts were then dried in an air oven (Shel Lab, USA) at 40 °C to remove the remaining co-solvent. All extracts were cooled at room temperature and placed in a desiccator before being gravimetrically weighed by using an analytical balance (± 0.0001 g) to determine the yields. The obtained extraction yield data was used to produce the OEC.

#### Scale-up process

**Figure 1:**
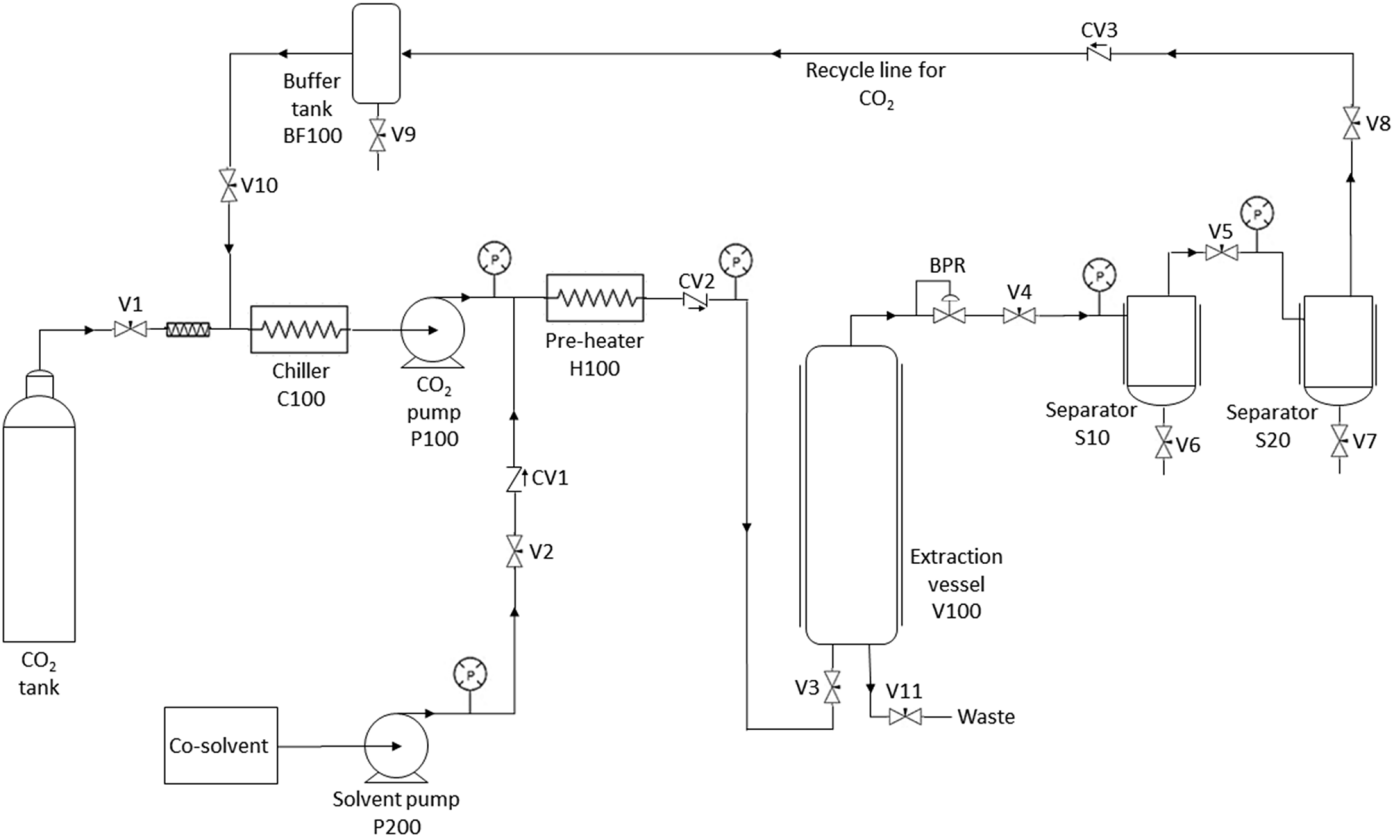
Schematic diagram of SFE extraction system with 5 L extraction vessel.

For scale-up validation extraction, the larger scale SFE system used was also an in house-built unit. It was equipped with one 5 L extraction vessel and two 300 mL separators displayed in series as shown in Fig. 1. The solvent mass to feed mass ratio (S/F) was the best scale-up criterion for this system based on the previous study for *P. niruri* extraction^32^. The behaviour of the process was predicted by using mathematical model, which was the modified Sovová model^34^. The scale-up prediction was investigated at both operating conditions, whereby the OEC obtained from laboratory-scale experiments was used as a reference. Thereafter, the scale-up parameters were inserted in the modified model and the best fitting was determined by using a correlation equation between dimensionless Reynolds number (Re) and mass transfer coefficient in fluid phase (*k*_*Ya*_). This scale-up procedure successfully predicted the scaling-up of overall extraction curves of *P. niruri* for feed capacity from 0.005 kg to 500 kg.

Therefore, the scale-up validation experiments for 0.5 kg feed capacity were conducted by using S/F scale-up criterion and similar methodology with the laboratory-scale. The operating conditions for scale-up experiment were similar to the laboratory-scale experiment, except for the solvent flow rate. The solvent flow rate was calculated by using the scale-up criterion, so that S/F was maintained from laboratory-scale (LS) to larger-scale SFE. The S/F values for PRE and ME curves of both operating conditions were presented in Table 3. PRE was the pre-extraction curve that was governed by CO_2_, meanwhile ME was the main extraction curve that was governed by water.

**Table 3:** S/F values that were maintained from laboratory-scale to larger-scale.

Operating condition	Extraction curve	S/F (kg solvent/kg sample)
L1	PRE	49.83
	ME	3.79
L2	PRE	43.73
	ME	6.61

PRE is pre-extraction curve and ME is main extraction curve.

**Figure 2:**
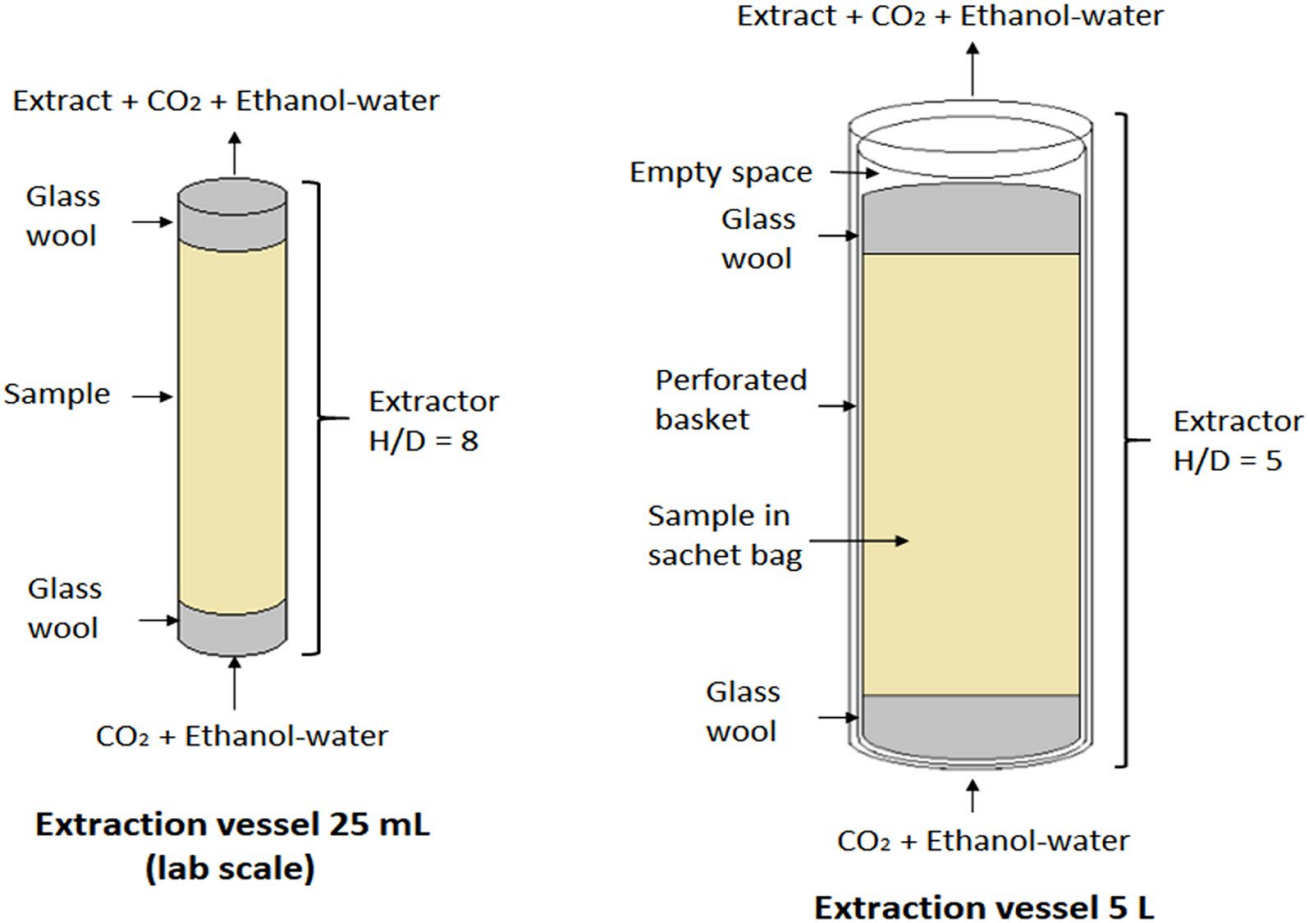
Internal configuration of extraction vessels at different SFE scales. H/D: ratio of height to diameter of the vessel, SS: stainless steel.

In laboratory-scale extraction vessel, the 5 g sample was inserted in a packed-bed with glass wool, which was placed at both ends. Meanwhile, for scale-up experiment, 0.5 kg sample was inserted in mesh bags and placed in a perforated basket with glass wool at both ends. Only then, the perforated basket was placed in the extraction vessel of scale-up SFE. The internal configurations for both extraction vessels are shown in Fig. 2.

### Quantification of bioactive compounds

Component contents were quantified by using a High-Performance Liquid Chromatography (HPLC) system 1100 series (Agilent Technologies, Germany). This HPLC system was equipped with an auto sampler and UV/Vis detector, with a reverse phase C18 column, Kinetex (250 × 4.6 mm, 4 µm). The mobile phase used were 0.1% phosphoric acid in water (solvent A) and acetonitrile (solvent B). Chromatographic method was conducted at 35 °C with a wavelength of 270 nm. The injected sample volume was 20 µL. Chromatographic peaks were identified by comparing HPLC retention time of gallic acid (GA), corilagin (CO), and ellagic acid (EA) standards.

### Content of ethanol residue

Ethanol quantification analysis was conducted by using ROA (organic acid H +) determination method by HPLC system 1200 series (Agilent Technologies, Germany), which was equipped with an auto sampler and refractive index detector. The column used was Rezex ROA (Phenomenex, USA) with 300 × 7.8 mm in size. Sulfuric acid in water, at 0.005 N (normality), was used as mobile phase with a flow rate of 0.6 mL/min. The chromatography method was performed at a temperature of 60 °C with injection volume of 20 µL. Five ethanol standard solutions were injected at different concentrations in the range of 0.5–4 mg/mL to produce the standard curve with linear regression. *P. niruri* extracts were dissolved in dimethyl sulfoxide (DMSO) in the range concentration of 1 to 5 mg/mL and ethanol chromatographic peak in the extracts was identified through comparison with the retention time of ethanol standard reference.

### Fish embryo toxicity test (FETT)

The scale-up validated L2 extract was selected to undergo toxicity test to evaluate the safety of the extract. The fish embryo toxicity test (FETT) is a toxicity test that only involves the use of fish embryos (not live fish); hence it does not require an ethical approval. However, the test was approved by the Malaysian Nuclear Agency as its experimental protocols were developed and approved by the Organisation for Economic Co-operation and Development (OECD). The experiments were performed in accordance with relevant guidelines and regulation based on the OECD guidelines for the Testing of Chemicals. The FETT experiments were also carried out in accordance with the Animal Research: Reporting of In Vivo Experiments (ARRIVE) guidelines and regulations.

The FETT was conducted in the laboratory of Medical Technology Department, Malaysian Nuclear Agency on zebrafish embryo (*Danio rerio*). It was based on the methodology reported by Thiagarajan et al.^35^, with little modifications. Zebrafish embryos were transferred to 96-well microplate by using a pipet at 24 h post fertilisation (24 hpf)^36^. The embryos were exposed to the *P. niruri* extract solution with different concentrations (10, 20, 30, 60, 130, 250, 500 and 1000 µg/mL). The toxicity of standard reference solutions (gallic acid, corilagin, and ellagic acid) were also investigated and compared with the extract solution. The embryos were also exposed to 1% propanol as positive control and distilled water as negative control. Analysis was conducted for three replications.

The embryo development was observed at 24, 48, 72 and 96 h post fertilisation (hpf). Examination on zebrafish embryos were conducted with a microscope by focusing on parameters, such as the embryos’ mobility, the presence of edema and also their heartbeat. The heartbeat of the embryos was calculated for 15 s and multiplied by 4 to get the total heartbeat per minute^37^. Results were also defined by the LD_50_ values, which was a lethal concentration or lethal dose at 50% that was determined through linear regression of mortality rate against log concentration.

### Economic analysis

In this section, economic and profitability analysis methodology by Peters et al.^38^ was used to determine the total capital investment (C_TC_), the total product cost, (C_TPC_), the return on investment (ROI), the payback period (PBP), the average annual net return (R_n,ave_), the net present value (NPV) and the discounted cash flow rate of return (DCFRR). The analysis was calculated for different scale of SFE units (5 L, 50 L and 500 L extraction vessel).

#### Process estimation and equipment cost

For estimation purposes, the SFE process was operated for 18 h with three daily shifts for 350 days, which made up 6,300 h of operation per year. This involved the cost of raw material covered for plant samples, carbon dioxide gas and co-solvent used (ethanol). Meanwhile, the cost of utility comprised of electricity and heat exchange agents used in the process. The cost of waste treatment was neglected because the plant residue of the SFE process could be commercialised as a by-product or incorporated into the soil. The identified economic parameters for this process was presented in Table 4.

**Table 4:** Required parameter for economic analysis.

Cost	Component	Unit	Value	Note/References
FI (fixed investment: equipment cost)	SFE 5 L	USD	74,146	This study
	Industrial unit of SFE 50 L	USD	567,025	Estimated cost*
	Industrial unit of SFE 500 L	USD	2,934,433	Estimated cost*
COL (operational labour)	5 L: 1 operator	USD/h	2.44	^39 (Minimum wage rate)^
	50 L: 5 operators			
	500 L: 15 operators			
CRM (raw material)	*Phyllanthus niruri*	USD/kg	13.41	HERBagus, Malaysia
	Carbon dioxide (CO_2_)	USD/kg	0.28	^40^
	Co-solvent (i.e.: ethanol)	USD/L	0.91	^41^
CUT (utility)	Electricity	USD/kWh	0.08	^42^
	Water	USD/m^3^	0.56	^43^
CWT (waste treatment)	–	–	–	–

*Calculated estimation cost by using Eq. (1).

For industrial unit of 50 L and 500 L, equipment estimation costs were calculated using the equation below:

1 $$C_{2} = C_{1} \left( {\frac{{Q_{2} }}{{Q_{1} }}} \right)^{n}$$

whereby *C*_2_ was the unknown equipment cost with capacity *Q*_2_ (L), *C*_1_ was the known base cost for equipment with capacity *Q*_1_ (L) and *n* was a constant depending on equipment type. Table 5 showed the base costs for 1 L SFE equipment, which was obtained from reference^25^.

**Table 5:** Base cost of different equipment for 1 L SFE system (*Source*: Pereira et al.^25^).

Equipment	*n*	Base cost/unit (USD)^*b*^
Storage tank	0.57	300
Jacketed extraction vessel	0.82	5540
CO_2_ pump	0.55	2470
Electric liquid pump	0.55	3920
Cooler	0.59	2080
Heater	0.59	820
Manometer	0	410
Block valve	0.6	220
Back-pressure valve	0.6	1780
Micro metering valve	0.6	1090
Flowmeter	0.6	700
Safety valve	0.6	310
Temperature controller	0.6	310
CO_2_ compressor	0.46	2200
Separator	0.49	1460
Piping, connectors, crossheads, mixers and splitters	0.6	3660
Structural material for supporting the equipment	0.6	4060
Total for SFE process (1 L) with co-solvent	–	39,790

*n* Constant depending on equipment type, *b* Based on 1 L SFE system.

#### *Total capital investment (C*_*TC*_*)*

C_TC_ is the total of fixed capital investment (C_FC_), working capital (C_WC_) and land capital (C_L_). Fixed investment was divided into two components, which were manufacturing fixed capital (direct cost) and non-manufacturing fixed capital (indirect cost). The working capital was in the range of 60 to 75% of total equipment cost. For solid–fluid processing plant, working capital was set at 75% from equipment cost^44^. For this study, the land capital for 5 L SFE unit was neglected because it was placed in a university’s research laboratory. Meanwhile, 50 L and 500 L SFE units were assumed to be constructed on two and five acres of industrial land. The land price was assumed to be USD 12/ft^2^. The identified economic components for calculation of C_TC_ were presented in Table 6. The components percentage were based on solid–fluid processing as reported by Peters et al.^38^.

**Table 6:** Economic component for total capital investment calculation for different scale of SFE units.

Category	*SFE* 5 L		*SFE* 50 L		*SFE* 500 L	
	A (%)	C (%)	A (%)	C (%)	A (%)	C (%)
**Fixed capital investment (C**_**FC**_**)**						
(i) Direct cost						
a. Purchased equipment, A	100		100		100	
b. Delivery, B	10		10		10	
c. Total equipment cost, C: A + B	–	100	–	100	–	100
d. Equipment installation	–	39	–	39	–	39
e. Instruments and controls	–	0	–	26	–	26
f. Piping	–	0	–	31	–	31
g. Electrical system	–	10	–	10	–	10
h. Building	–	0	–	29	–	29
i. Yard improvement	–	0	–	12	–	12
j. Services facilities	–	0	–	55	–	55
(ii) Indirect cost						
a. Engineering and supervision	–	32	–	32	–	32
b. Construction expenses	–	0	–	34	–	34
c. Legal expenses	–	4	–	4	–	4
d. Contractor’s fee	–	19	–	19	–	19
e. Contingencies	–	37	–	37	–	37
Working capital (C_WC_)	–	75	–	75	–	75
Land capital (CL)	–	–	–	USD 12/ft^2^*	–	USD 12/ft^2^*

*Estimated land price in Malaysia based on several website searches.

#### *Total product cost (C*_*TPC*_*)*

The total product cost is generally divided into two, which are the manufacturing costs (MC) and also the general expenses (GE). MC is a cost that directly related to the manufacturing operation of a process plant. It could be categorised into three parts; direct manufacturing cost (DMC), fixed charges (FC) and plant overhead cost (PO). Meanwhile, GE may be classified into administrative expenses, distribution and marketing expenses and research and development expenses (R&D). The components in C_TPC_ and their assumption factors were shown in Table 7.

**Table 7:** Assumption factor for components in total product cost.

Total product cost		Component	Assumption
MC	DMC	Raw materials (C_RM_)	From calculation
		Utilities (C_UT_)	10% C_TPC_
		Operating labour (C_LB_)	From calculation
		Management and supervision (C_SV_)	15% C_LB_
		Maintenance and repair (C_MT_)	5% C_FC_
		Operational supplies C_SUP_)	15% C_MT_
		Laboratory charges (C_LAB_)	7% C_LB_
		Royalty (C_ROY_)	1% C_TPC_
	FC	Local taxes (C_TAX_)	2% C_FC_
		Insurance (C_INS_)	1% C_FC_
		Financing (C_FIN_)	2% C_FC_
	PO	Total C_LB_, C_SV_ and C_MT_	60% (C_LB_ + C_SV_ + C_MT_)
GE		Administrative costs	20% (C_LB_ + C_SV_ + C_MT_)
		Distribution and selling cost	5% C_TPC_
		Research and development (R&D)	4% C_TPC_
		Extract safety research	4% C_TPC_

*MC* Manufacturing cost, *DMC* Direct manufacturing cost, *FC* Fixed charges, *PO* Plant overhead, *GE* General expenses, *C*_*TPC*_ Total product cost, *C*_*FC*_ Fixed capital investment.

#### Depreciation

The method used to calculate annual depreciation charge was the straight-line method. Property value was assumed to decrease linearly with time over the service life of the processing plant. The annual depreciation was calculated by using the equation below based on the analysis that had been made:

2 $$A_{D} = \frac{{C_{FC} - S}}{n}$$

whereby *A*_*D*_ was the annual depreciation charge, *C*_*FC*_ was the fixed capital investment, *S*_*C*_ was the scrap value for a plant or equipment at the end of service life and *n* was the service life. In this study, the project service life was set at 15 years with 25% tax rate and the scrap value was set to zero.

### Profitability analysis

Return on investment (ROI) is the ratio of annual profit to the total capital investment. The SFE process was considered as medium risk investment, whereby the rate of return was in the range of 16% to 24%. This rate was known as minimum acceptable rate of return (MARR or m_ar_). For this process, the m_ar_ value was set at 24% (0.24) and compared to the ROI value. In general, it is profitable for a project if the ROI value is larger than m_ar_. Meanwhile, the PBP is the investment recovery period or time needed to acquire the investment cost calculated from the ROI as the base^45^. For this study, the PBP value was compared with the reference PBP, which was 2.87 years.

Other than that, NPV is the difference between the current value of cash inflows and the current value of cash outflows over a period of time. It was equal to the cumulative discounted cash flow value at the end of a project’s life span, which was 15 years for this process. The DCFRR or also known as internal rate of return (IRR), presented the average intrinsic profitability for a project. It is also defined as the interest rate that gives a net present value of zero^45–47^. It shows that the higher the DCFRR value, the more interesting a project due to a more promising profitable return. Therefore, this project is profitable if:

1. ROI value is larger than m_ar_
2. PBP value is smaller than PBP_ref_
3. R_n,ave_ value is positive
4. NPV value is positive
5. DCFRR is larger than m_ar_

## Results and discussion

### Overall extraction curve (OEC)

**Figure 3:**
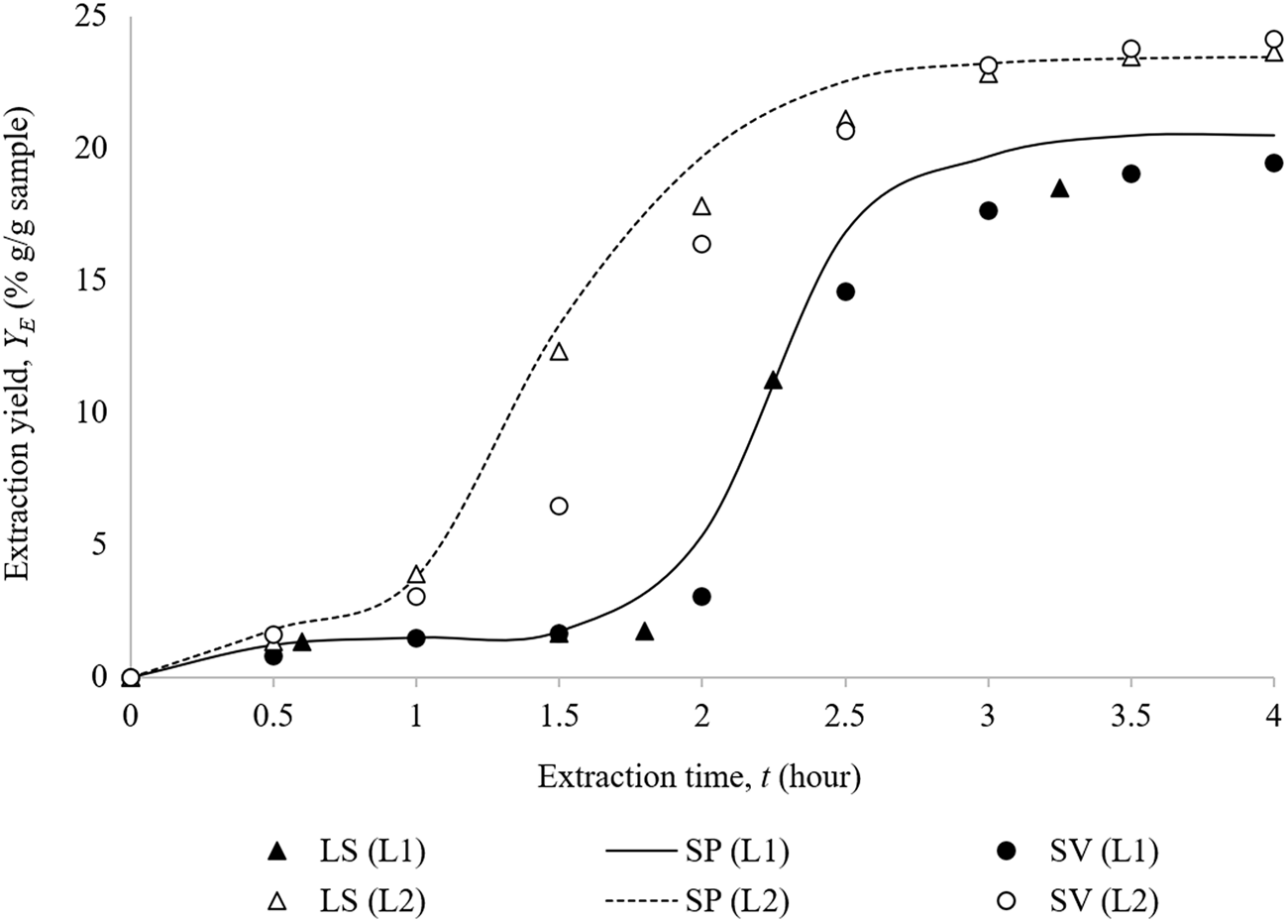
Comparison of OEC for laboratory-scale experiment (LS) with OEC for scale-up prediction (SP) and scale-up validation experiment (SV) at L1 and L2. LS and SP data were obtained from previous study^32^. L1: P = 200 bar, T = 60 °C, co-solvent = 50% ethanol–water with 10% (v/v) concentration, L2: P = 262 bar, T = 80 °C, co-solvent = 30% ethanol–water with 13% (v/v) concentration.

The OEC comparison between model prediction that was obtained from the previous study^32^, and validation experiment for 0.5 kg feed capacity at both L1 and L2 operating conditions were presented in Fig. 3. Both OECs were also compared to the OECs from laboratory-scale experiments. The results showed that the scale-up criterion by maintaining the S/F was successful for obtaining a similar shape of laboratory-scale OECs. Two different curves were observed in all OECs. This was due to high content of co-solvent, which caused the liquid separation in the vessel. The first curve (pre-extraction or PRE) was obtained from the extraction with pure CO_2_. meanwhile the second extraction curve (main extraction or ME) started with the appearance of hydrophilic compounds in the extract. It was hypothesised that, the sudden increase of extraction yield in this unusual second extraction curve was caused by the change in the governing solvent from CO_2_ to water^17^, which its behaviour was likely to pressurised solvent extraction.

The total extraction yield for modelling prediction and validation experiment was compared and presented in Table 8. Results showed that the yield obtained from scale-up validation experiment was comparable to the predicted yield, with errors of 5.13% for L1 and 2.92% for L2. Meanwhile, the average absolute relative deviation (AARD) percentage of overall extraction curve error for both operating conditions were 13.90% and 14.20%, respectively. The high value of AARD was due to slower production of the second curve (main extraction curve, ME), if compared to the predicted curve. For L1 operating condition, the second curve of ME was predicted to be produced at the fourth fraction (1.5–2 h of extraction time). However, the second curve with brownish fraction for validation experiment was produced 30 min later, at extraction time of 2–2.5 h (fifth fraction). The difference caused a higher error for that fraction as shown in Table 8.

**Table 8:** Fraction and cumulative extraction yield for scale-up prediction and validation of 0.5 kg feed sample.

Operating condition	Fraction	Cumulative yield, *Y*_*E*_ (% g/g sample)		Error (%)
		Prediction*	Validation	
L1	1	1.23	0.78	36.81
	2	1.51	1.48	2.00
	3	1.74	1.67	4.46
	4	5.37	3.05	43.18
	5	16.81	14.58	13.26
	6	19.68	17.63	10.40
	7	20.47	19.04	6.99
	8	20.48	19.43	5.13
	AARD OEC (%)	4.44	13.90	–
L2	1	1.81	1.59	12.50
	2	3.81	3.07	19.59
	3	13.35	6.46	51.63
	4	19.69	16.37	16.88
	5	22.53	20.67	8.28
	6	23.20	23.14	0.28
	7	23.39	23.77	1.59
	8	23.45	24.13	2.92
	AARD OEC (%)	6.13	14.21	–

L1: P = 200 bar, T = 60 °C, co-solvent = 50% ethanol–water with 10% (v/v) concentration, L2: P = 262 bar, T = 80 °C, co-solvent = 30% ethanol–water with 13% (v/v) concentration.

*Prediction data were obtained from previous study^32^.

A similar result was observed for L2 operating condition, whereby the validation experiment produced the second curve 30 min slower (during the fourth fraction) than the predicted curve (during the third fraction). This was possibly due to the different particle sizes used for both scales. A very small particle size was probably not suitable to be used in larger-scale experiments as it would cause a significant channelling effect (biomass accumulation leading to pipeline blockage)^48^. Therefore, a larger particle size was used in scale-up validation experiment to decrease the channelling effect. According to Fiori et al.^49^, for samples with a larger particle size, the extraction yield decreased after a certain time because of the delayed extraction of solute that bonded to larger particle size. Furthermore, based on the internal configuration for larger scale vessel (Fig. 2), there was some empty space or void volume between mesh bags, perforated basket and also the extraction vessel. According to Le Floch et al.^50^, void volume should be reduced so that no additional time was required to transfer out the extraction yield from the vessel. It showed that void volume effect could delay the mass transfer, especially for heavier components. Previous study also reported the unsatisfactory extraction yield was due to the effect of void volume^51,52^.

Modelling results from the scale-up prediction study for *P. niruri* extraction showed that the difference in height and diameter ratio of the vessel (H/D) for different scales did not influence the prediction of extraction curve^32^. However, it should be taken into consideration in further studies, as the bed geometry differences between the two systems could alter the diffusion, increased the axial diffusion contribution and eventually affecting the mass transfer. Overall, the obtained fractions were comparable to the laboratory-scale fractions. It showed that the 5 L SFE unit was able to extract 0.5 kg of *P. niruri* with CO_2_ and ethanol–water co-solvent by using the S/F scale-up criterion at the given operating conditions. However, because the fractions for the validation experiment were obtained slower than predicted, the bioactive compounds quantification for their extracts should be conducted and compared with the laboratory-scale extracts (0.005 kg).

### Component yield

**Figure 4:**
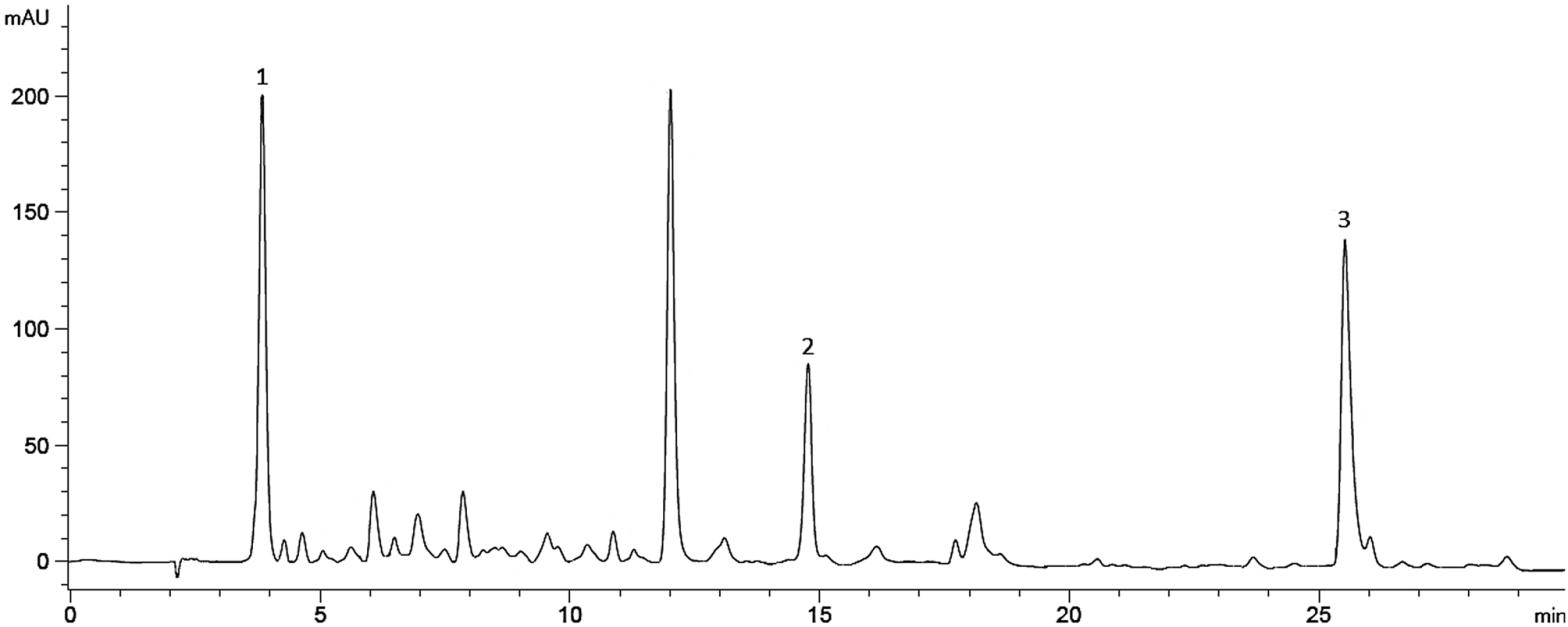
Chromatographic profile for the fourth fraction of L2 validated scale-up extract. Gallic acid (1), corilagin (2), ellagic acid (3).

From the HPLC analysis, three targeted bioactive compounds were identified in validated scale-up extract as shown in Fig. 4 (for L2 extract). It showed that good peak absorbance and separation could be achieved by gradient elution of acetonitrile–water system at UV wavelength of 270 nm. The initial identified component was gallic acid with retention time of 3.84 min, followed by corilagin and ellagic acid with retention time of 14.85 min and 25.41 min respectively. These three components had been identified in *P. niruri* in previous studies^53–55^.

**Figure 5:**
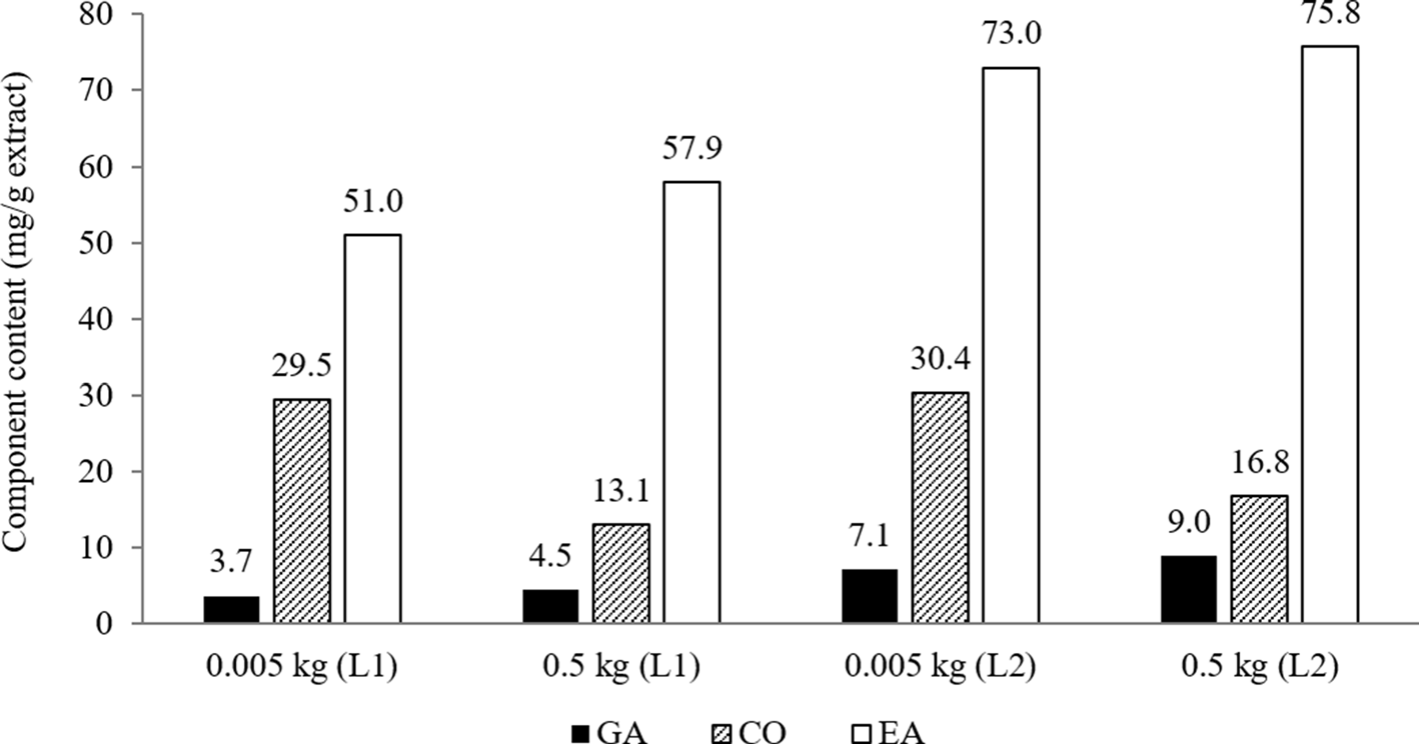
Total component content in laboratory-scale extract (0.005 kg sample) and scale-up extract (0.5 kg sample) for L1 and L2 operating condition. GA: gallic acid, CO: corilagin, EA: ellagic acid.

Furthermore, the component contents for laboratory-scale extract and scale-up extract were also compared as presented in Fig. 5. From the figure, the content of the three components for both operating conditions were relatively higher compared to the commercial Nova HEPAR-P standardized *P. niruri* extract (gallic acid: 2.1 mg/g extract, corilagin: 26.4 mg/g extract, and ellagic acid: 41.7 mg/g extract) as reported by Markom et al.^18^. It could be concluded that the L1 and L2 extracts were enriched with the bioactive compounds and they were better than the commercial standardised extract. Results also showed that gallic acid and ellagic acid contents in scale-up extracts were higher than the laboratory-scale extracts. However, corilagin content showed an opposite result, whereby the content in laboratory-scale extract was higher. This was possibly due to different particle sizes used for both scales as discussed on the overall extraction curve before.

Another possibility for this result was that corilagin had been hydrolysed to gallic acid and ellagic acid. Yisimayili et al.^56^ reported that corilagin could undergo the hydrolysis process to form gallic acid, ellagic acid and M3 (hydrolysed ellagitannin metabolite). This hydrolysis process decreased the corilagin content, but also increased the gallic acid and ellagic acid. Although the validation extraction was conducted by using the same S/F ratio with laboratory-scale, the solvent residence time in the scale-up vessel was much longer due to its larger volume. The increase of solvent residence time (hydrolysis time) probably increased the corilagin hydrolysis rate. As mentioned by Singh and Bishnoi^57^, hydrolysis time was one of the significant parameters in the optimisation of enzyme hydrolysis for ethanol production from yeast.

Moreover, the difference of distribution uniformity for supercritical fluid (SCF) in different extraction vessels could be one of the factors, which differed the component contents for both SFE scales. The more compact internal configuration for laboratory-scale vessel caused a more uniformed SCF distribution if compared to the scale-up vessel (as shown in Fig. 2). The difference in SCF distribution probably influenced the solubility and internal mass transfer for corilagin. Moreover, the placement of the sample in mesh bags and perforated basket might decrease the bed compactness and the bulk density of the sample, whereby the channelling effect could occur.

### Product safety: content of ethanol residue

Both dried validated scale-up extracts were analysed by HPLC to determine the total content of ethanol residue. This analysis was conducted to determine whether the extracts were safe or not to be consumed orally. The total content of ethanol residue for validated scale-up extract at both L1 and L2 operating conditions were presented in Table 9. The results showed that the content ethanol residue for L1 extract was higher than the L2 extract. This was because higher co-solvent concentration was used for experiment at L1 operating condition, which was 50% (v/v) ethanol in water, compared to 30% (v/v) ethanol in water for L2 operating condition. Other than that, the extraction temperature for L2 was higher (80 °C) than the extraction temperature for L1 (60 °C). The boiling point for ethanol is 78.3 °C, therefore the high temperature for L2 would probably evaporate the ethanol faster than the lower temperature of L1.

**Table 9:** Ethanol residue content in dried *P. niruri* extract.

Extract	Total content (% g/g extract)	Total yield (% g/g sample)	Weight (mg)
L1	1.98	0.38	19.22
L2	1.56	0.37	18.85

L1: P = 200 bar, T = 60 °C, 50% ethanol–water co-solvent, L2: P = 262 bar, T = 80 °C, 30% ethanol–water co-solvent.

Table 9 also showed that the total content of ethanol residue for both extracts were still high (1.98% and 1.56%), when compared to the ethanol percentage limitation in the final product to fulfil the halal requirement. The allowable ethanol percentage limit (industrial ethanol) in the final product for halal food in Malaysia is 0.5%^58^. The high content of ethanol residue in both extracts were probably due to imperfect drying process. As mentioned by Rasit et al.^59^, a prolonged heating could destroy certain thermolabile phytochemical compounds when exposed to higher temperature. In this study, a simple drying process was executed by using an oven at a low temperature (40 °C) to prevent thermal degradation on the bioactive compounds. However, the low temperature probably caused the ethanol not to vaporise completely. Hence, a further study to emphasise a more suitable drying method is very crucial to achieve the halal status for the final product. Moreover, other factors that could affect the ethanol residue, such as flushing time, time from drying to analysis and the exposure to air should be considered in further studies.

However, these *P. niruri* extracts were still safe to be used since a total ethanol residue of 50 mg per day is acceptable for human health^60^. The total ethanol in *P. niruri* extracted at both operating conditions were 19.22 mg and 18.85 mg, respectively, which were much lower than the 50 mg limitation. Aside from that, according to the Food and Drug Administration (FDA) classification, ethanol is a GRAS (generally recognised as safe) solvent^61^, and it was often used in food and pharmaceutical industries.

### Product safety: toxicity of the extract

Toxicology test on the extract of herbal plant is crucial to ensure its safety and implication for its usage as medicine and health supplements. It is crucial to evaluate the risks or potential hazards to human through laboratory animals. In this study, the validated scale-up extract at L2 operating condition was selected to be analysed for its safety level by using the FETT on *Danio rerio* embryo or commonly known as zebrafish.

**Figure 6:**
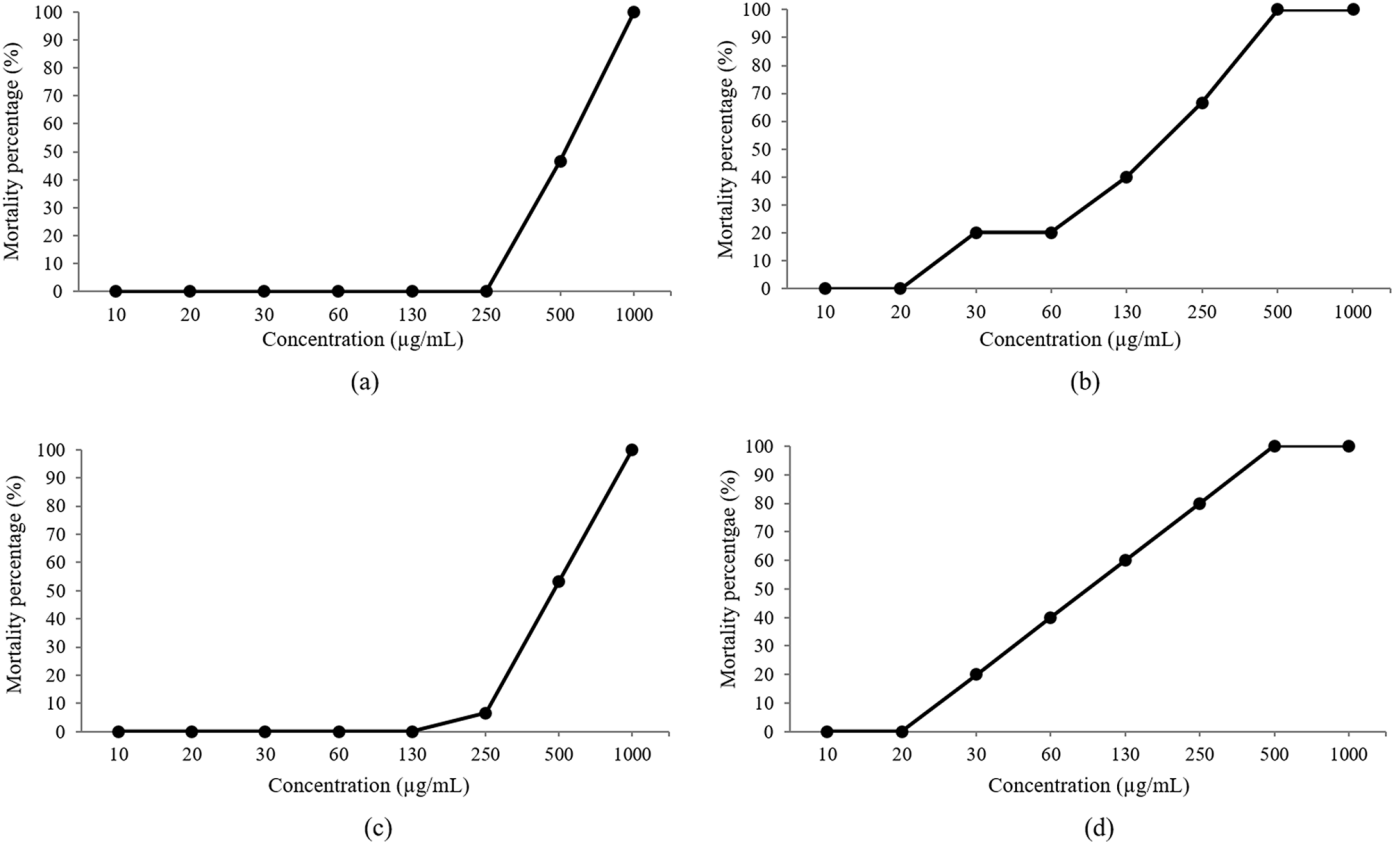
Average embryo mortality percentage against concentration of extract and standard reference solution after 96 h of observation: (**a**) *P. niruri* extract (**b**) gallic acid, (**c**) corilagin, (**d**) ellagic acid.

Based on the results in Fig. 6, after 96 h of observation, the embryo mortality rate increased along with the concentration of extract and standard reference solution. The mortality percentage was presented by unfertilised and dead embryos, which included coagulation of embryos, non-detachment of the tail, irregularities in somite formation and also lack of heartbeat^62^. The zebrafish embryos in *P. niruri* extract showed the highest survival rate compared to other standard references (gallic acid, corilagin and ellagic acid), whereby embryos’ mortality rate could only be seen at high extract concentration, which was 500 µg/mL and above. Meanwhile, gallic acid and ellagic acid showed a high mortality rate at concentrations as low as 30 µg/mL.

**Figure 7:**
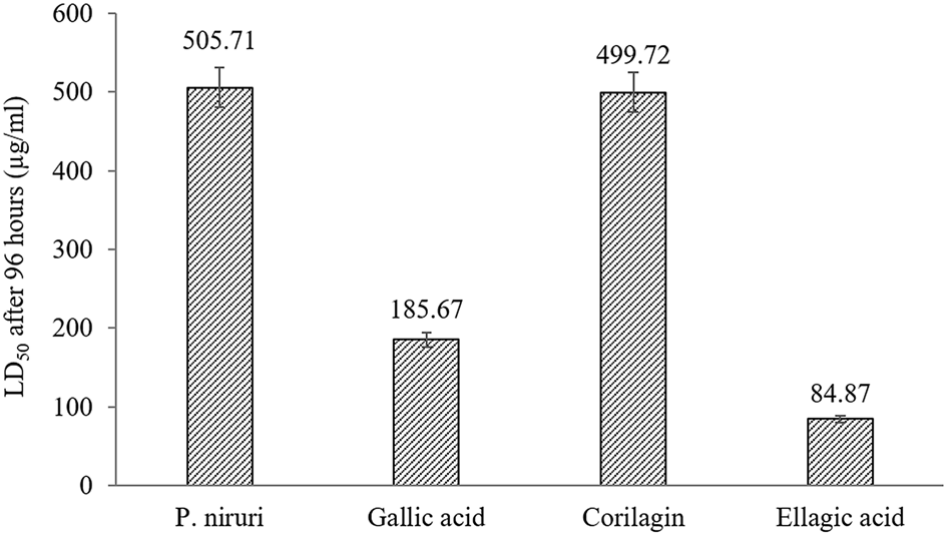
LD_50_ values for *P. niruri* extract and standard reference.

To evaluate the toxicity level of *P. niruri* extract, the median lethal dose (concentration) values, which are known as LD_50_ or LC_50_, were determined. These values were obtained by using an online LD_50_ calculator^63^ and were shown in Fig. 7. Generally, a higher value LD_50_ showed a low level of toxicity because a higher dose/concentration was needed to produce 50% mortality rate^64^. The LD_50_ value for *P. niruri* extract was the highest (505.71 µg/mL) compared to the other three standard references. It showed that *P. niruri* extract with concentration lower than this value was safe against the zebrafish embryos.

Meanwhile, ellagic acid showed the highest level of toxicity with a low LD_50_ value, which was 84.87 µg/mL. The OECD and European Chemicals Bureau (ECB) categorised the toxicity of pollutants against zebrafish into three ranges of LC_50_ values, which were harmful (10 µg/mL < LC_50_ < 100 µg/mL), toxic (1 µg/mL < LC_50_ < 10 µg/mL) and very toxic (LC_50_ < 1 µg/mL)^65^. Based on these categories, *P. niruri* extract, gallic acid and corilagin were in the safe category. However, ellagic acid was in the first category, which was harmful.

Other than determining the LD_50_ value, the observation on morphological deformities was conducted at 24 h, 48 h, 72 h and 96 h post fertilisation. The results were presented in Table 10. It was observed that for negative control, *P. niruri* extract and corilagin, the embryos were well-developed and exhibited no abnormalities, from the initial 96 h of treatment in the media. At the end of 96 h cycle, the embryos had achieved their full form and moved around the well. Meanwhile, there were some morphological changes that occurred to the fish embryos, which were treated in selected doses of gallic acid, ellagic acid and 1% propanol (positive control).

**Table 10:** Observation on zebrafish embryos from 0 to 96 h post fertilisation.

Extract/standard reference	Concentration* (µg/mL)	Cycle stage (h)				
		0 (Fertilised embryo)	24 (Pharyngula stage)	48 (Hatching stage)	72 (Early larvae stage)	96 (Full form stage)
Distilled water (negative control)	–		No abnormality	No abnormality	No abnormality	Full form (normal movement)
*P. niruri*	500		No abnormality	No abnormality	No abnormality	Full form (normal movement)
Gallic acid	250		Slower movement	Slower movement	Slower movement	Slower movement
Corilagin	500		No abnormality	No abnormality	No abnormality	Full form (normal movement)
Ellagic acid	250		Slight edema	Slight edema	Slight edema	Slight edema (imbalance)
1% Propanol (positive control)	–		Edema presence	Edema presence	Edema presence	Edema enlargement (immobile)

*Selected concentration for embryos observation under microscope.

The zebrafish embryo treated in 250 µg/mL gallic acid was observed to be slower than the normal after 24-h period and this condition continued to be observed at the 96 h. Whereas, the zebrafish embryo treated in ellagic acid at the mentioned dose developed slight edema after 24-h period and at the end of the 96 h cycle. It seemed that the embryo had problems balancing itself in which the larvae appeared to tip to the side. For the embryo in 1% propanol, edema was presented and in the final period of 96 h, the embryo exhibited enlargement of the edema and was rendered immobile. This condition showed that 1% propanol was toxic against zebrafish.

The observed abnormalities in terms of mortality rate and general morphology showed that *P. niruri* extract below 500 µg/mL was not harmful against the zebrafish embryo. Moreover, Asare et al.^66^ reported that there was no observation on acute toxicity against laboratory rat that was given *P. niruri* leaf extract at dose of 2000 mg/mL, in which it was safe to be used as an alternative treatment. Other than the three mentioned bioactive compounds, *P. niruri* also contained other beneficial components like flavonoid (condensed tannins). Flavonoids such as quercetin, rutin, (+)-catechin, (−)-epicatechin, (+)-gallocatechin and (−)-epigallocatechin had been identified in *P. niruri* extracts as reported by Markom et al.^18^. Flavonoid is an antioxidative compound that could remove free radicals by acting as a neutralisation agent^67^. The absence of flavonoid would cause the oxidative stress, which would harm the molecular cell indirectly and finally lead to the low survival rate of zebrafish embryos^68^.

**Figure 8:**
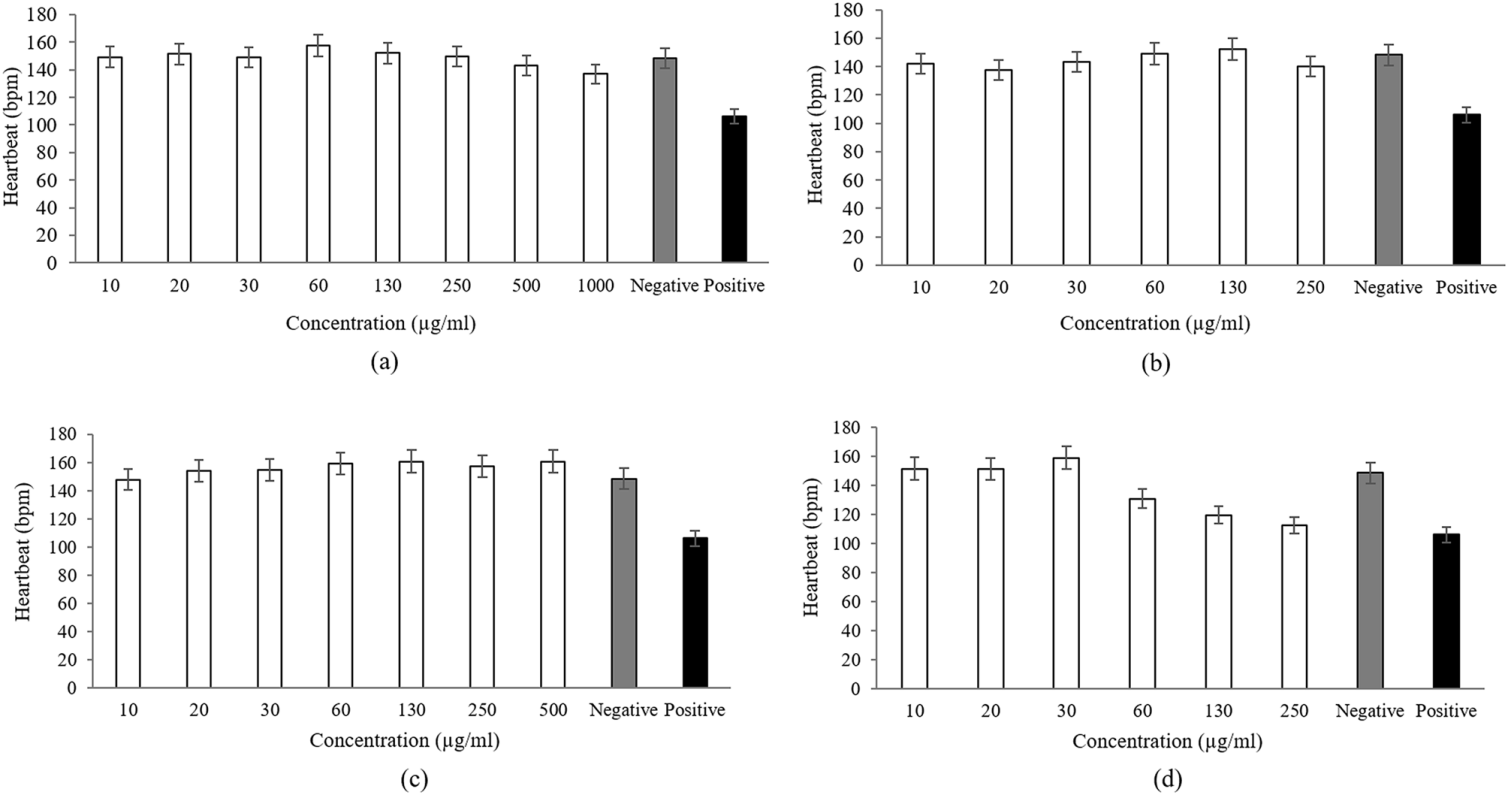
Zebrafish heartbeat after 96 h in different concentration of extract/standard reference solution: (**a**) *P. niruri* extract, (**b**) gallic acid, (**c**) corilagin and (**d**) ellagic acid.

The heartbeat for a developed zebrafish begins at 36 h post fertilisation^69^, whereby the normal heartbeat was between 120 and 180 beats per minute (bpm)^35^. The observation on the fish heartbeat after 96 h of treatment at different concentrations of extracts/standard references were presented in Fig. 8. The heartbeat for negative and positive controls were also compared in this figure. From Fig. 8a, it showed that heartbeat of embryos treated in *P. niruri* extract with concentration until 1000 µg/mL were in the normal range, similar with the negative control. Meanwhile, the positive control showed a slow average heartbeat which was under the normal range.

For gallic acid, Fig. 8b showed an increment of heartbeat with increasing concentration up to 130 µg/mL and then decreased at concentration of 250 µg/mL, but still in the normal range. Concentration higher than this value recorded the death of the embryos (no heartbeat detected). Besides, Fig. 8c showed the heartbeat increment (in the normal range) at a concentration below 500 µg/mL. Finally, the heartbeat of the fish was in the normal range when treated in ellagic acid with a concentration below 60 µg/mL as shown in Fig. 8d. At concentrations between 130 and 250 µg/mL, the heartbeat was slower (under the normal range) but did not cause the death of the fish.

For zebrafish embryos that were treated in gallic acid and ellagic acid at a concentration of 250 µg/mL and above, there was no heartbeat recorded, which indicated that death had occurred. The changes in the heartbeat were probably due to the cardiac function that was affected by the under-developed pericardium and heart. This condition led to the abnormal heartbeat and failure of circulation, which finally caused deformity to body development due to nutrient deficiency^70^. Therefore, the deficiency experienced by the embryos would lead to their deaths.

In terms of heartbeat abnormality, mortality percentage and observation on the general morphology from the FETT analysis showed that the *P. niruri* extract was not harmful to the fish embryos at low concentration (below 500 µg/mL). However, a different finding was obtained by Lamban et al.^71^, whereby it was reported that the extract of *P. niruri* leaves by hot water maceration method was embryo-toxic and teratogenic against zebrafish. The method of hot water maceration probably damaged the flavonoid content in the extract. As mentioned before, flavonoid is an important component as an antioxidant and its absence will cause oxidative stress and damage the fish molecular cell indirectly.

Additionally, it was known that the extraction of flavonoid was more efficient at low temperature as high temperature degraded the component^67^. This condition also showed that the SFE extraction was more effective compared to the conventional extraction. Although the SFE extract of *P. niruri* was categorised as safe according to the proposed category by the OECD, the extract still showed the toxicity effect at high concentration as evaluated in the embryo’s mortality percentage. Therefore, it is very important to conduct a further study on phytochemical screening to identify the specific component that causes the toxicity effects by this plant.

### Economic evaluation and profitability analysis

The product selling price of *P. niruri* extract was estimated from the market price of Nova HEPAR-P, a product by Nova Laboratories Sdn. Bhd, Malaysia. In 2020, the market price for this product was USD 34.45/bottle, which contained 60 capsules of standardised *P. niruri* extract (250 mg per capsule). By considering the extract quality of this process, which was enriched by bioactive compounds (higher component content compared to the commercial Nova HEPAR-P), the marketing strategy for this product was focused on pharmaceutical or healthcare products. Therefore, the selling price for *P. niruri* extract from this process was fixed at USD 1,709/kg.

The economic evaluation for scale-up prediction data and scale-up validation experiment for feed capacity of 0.5 kg at both L1 and L2 operating conditions were compared in Table 11. The results showed that the value of scale-up validation experiment for L1 was higher than the scale-up prediction due to lower extraction yields. Meanwhile for L2, the cost of manufacturing (COM) value for scale-up validation was lower due to higher extraction yields. The higher production for scale-up validation experiment simultaneously increases its sales and gross profit. Therefore, from the economic point of view, it could be considered that the L2 operating condition was better than L1.

**Table 11:** Economic evaluation of 0.5 kg feed capacity for L1 and L2 operating conditions by using data from scale-up prediction and scale-up validation.

Economic parameter	L1		L2	
	Prediction	Validation	Prediction	Validation
Production (kg/year)	112.79	102.00	123.09	126.69
Operation cost (USD/year)	92,683	92,683	92,683	92,683
COM (USD/kg extract)	832	911	747	725
Sales (USD/year)	192,683	173,171	209,756	217,073
Gross profit (USD/year)	97,561	80,488	117,073	124,390

L1: P = 200 bar, T = 60 °C, 50% ethanol–water co-solvent, L2: P = 262 bar, T = 80 °C, 30% ethanol–water co-solvent, COM: cost of manufacturing.

For profitability analysis, the annual depreciation, ROI, PBP, R_n,ave_, NPV and the DCFRR were predicted for three different extractor volumes (5 L, 50 L and 500 L). For the SFE system of 5 L extractor, the validated scale-up data of 0.5 kg feed was used. Meanwhile, the scale-up prediction data of the estimated annual full capacity was used for the SFE system of 50 L and 500 L. The summary of profitability analysis for the three SFE scales were shown in Tables 12 (LI) and 13 (L2).

**Table 12:** Summary of economic analysis of different SFE system for L1 operating condition.

Component	SFE 5 L	SFE 50 L	SFE 500 L
	Scale-up validation (S/F)	Scale-up prediction	Scale-up prediction
Total capital investment	USD 258,537	USD 4.20 million	USD 18.89 million
Annual production	105 kg	2,272 kg	11,200 kg
Annual total product cost (C_TPC_), at full capacity (except for SFE 5 L)	USD 102,439	USD 1.16 million	USD 5.47 million
Total depreciation	USD 26,806	USD 315,841	USD 1.63 million
Return on investment, ROI	0.25	0.50	0.56
Average net return, R_n,ave_	USD 248	RM 1.03 million	RM 5.70 million
Payback period, PBP	2.74 year	1.29 year	1.30 year
Net present value, NPV	USD 80,488	USD 4.58 million	USD 25.46 million
Discounted cash flow rate of return, DCFRR	0.32	0.49	0.54

**Table 13:** Summary of economic analysis of different SFE system for L2 operating condition.

Component	SFE 5 L	SFE 50 L	SFE 500 L
	Scale-up validation (S/F)	Scale-up prediction	Scale-up prediction
Total capital investment	USD 258,537	USD 4.20 million	USD 18.89 million
Annual production	127 kg	2,443 kg	12,080 kg
Annual total product cost (C_TPC_), at full capacity (except for SFE 5 L)	USD 100,000	USD 1.12 million	USD 5.28 million
Total depreciation	USDD 26,806	USD 315,841	USD 1.63 million
Return on investment, ROI	0.37	0.56	0.63
Average net return, R_n,ave_	USD 29,268	USD 1.26 million	USD 6.92 million
Payback period, PBP	1.96 year	1.16 year	1.17 year
Net present value, NPV	USD 185,366	USD 5.45 million	USD 29.95 million
Discounted cash flow rate of return, DCFRR	0.41	0.53	0.59

Results in Tables 12 and 13 showed that the annual depreciation values of different SFE units were similar for both L1 and L2 operating conditions. This was because the calculation only involved fixed capital investment for a system, which was not affected by other costs. Results also showed that the obtained ROI values at both operating conditions for different SFE systems were larger than m_ar_ value (0.24). Moreover, all SFE systems also showed a positive value of the average net return, R_n,ave_. The positive value indicated that the project’s cash flow was larger than the total needed to pay-back the investment, and to obtain the return that fulfilled the m_ar_ limitation. Other than that, the PBP is the best variable to show the profitability of a process^40^. For food industry in general, the required pay-back period was approximately two years^72^. All the obtained PBP values in Tables 12 and 13 were smaller than PBP_ref_ (2.87 years). Hence, it could be considered that this process fulfilled the requirement of a profitable project for different scales of SFE.

In addition, the NPV was considered as the most significant index for economic evaluation^46^. The positive NPVs for all SFE systems in Tables 12 and 13 showed that this process was economically feasible if the *P. niruri* extract was commercialised at selling price which was higher than USD 1,709/kg extract. Besides, a project could be approved when the calculated DCFRR was larger or equal to m_ar_ value (minimum value of *ROI*)^47^. In this study, the DCFRR value for different SFE systems were larger than m_ar_ value (0.24). Therefore, it could be considered that this project was profitable because it fulfilled both NPV and DCFRR requirements.

## Conclusion

In this study, the validation experiment of S/F scale-up criteria was successfully conducted, whereby the OEC obtained was similar to the OEC of laboratory-scale. The scale-up experiment could extract the three main components of *P. niruri,* namely gallic acid, corilagin and ellagic acid as reported in previous laboratory-scale studies. It indicated that the S/F scale-up criterion could predict the most satisfying OEC reproducibility and obtained an almost similar quality of extract to the laboratory-scale extract. Furthermore, the safety of the scale-up extract was successfully evaluated, whereby the content of ethanol residue in the dry extract was low and safe to be consumed. The treatment of zebrafish embryo in *P. niruri* extract through FETT showed a low toxic effect on the overall parameters such as survival rate, LD_50_ value, general morphological observation and the heartbeat rate. Additionally, the profitability analysis indicated the process feasibility of *P. niruri* extraction for the three scales of SFE system (5 L, 50 L and 500 L). Therefore, this economic analysis provided the information for deciding whether a scale-up project was feasible or not, which was very important for a more systematic and accurate scale-up approach.
