# Publisher Correction: Scale-up approach for supercritical fluid extraction with ethanol–water modified carbon dioxide on *Phyllanthus niruri* for safe enriched herbal extracts

**DOI:** 10.1038/s41598-021-96669-x

**Published:** 2021-08-20

**Authors:** Norsyamimi Hassim, Masturah Markom, Masli Irwan Rosli, Shuhaida Harun

**Affiliations:** Department of Chemical and Process Engineering, Faculty of Engineering and Built Environment, Universiti Kebangsaan Malaysia, 43600 UKM Bangi, Selangor Malaysia

Correction to: *Scientific Reports* 10.1038/s41598-021-95222-0, published online 4 August 2021

The original version of this Article contained an error in the Acknowledgements section.

“The authors would like to thank the Ministry of Agriculture, Malaysia (NKEA Research Grant Scheme, NH1113P008-2) and Universiti Kebangsaan Malaysia (ETP-2013-062 and GUP-2016-053) for the financial support.”

now reads:

“The authors would like to thank the Ministry of Agriculture, Malaysia (NKEA Research Grant Scheme, NH1113P008-2) and Universiti Kebangsaan Malaysia (ETP-2013-062 and GUP-2019-009) for the financial support.”

The original Article has been corrected.

